# Plant-Mediated Nanomaterials for Photoprotection: Mechanistic Insights, Current Advances, and Future Perspectives

**DOI:** 10.3390/nano16160988

**Published:** 2026-08-10

**Authors:** Nahid Moradi, Richard Bright

**Affiliations:** 1 Biotechnology Research Center, Shiraz University of Medical Sciences, Shiraz P.O. Box 71348-14336, Iran; nahidmoradi.phd@gmail.com; 2 College of Medicine and Public Health, Flinders University, Bedford Park, Adelaide, SA 5042, Australia

**Keywords:** plant-derived nanoparticles, green synthesis, nanocomposites, UV protection, photoprotection, herbal nanomaterials, zinc oxide nanoparticles, skin cancer prevention

## Abstract

Ultraviolet (UV) radiation is a major environmental factor contributing to photoaging, oxidative stress, inflammation, DNA damage, and photocarcinogenesis. Conventional UV filters, although widely used in sunscreen formulations, are associated with limitations including photoinstability, photocatalytic ROS generation, potential toxicity, and environmental concerns. In recent years, plant-mediated nanomaterials have emerged as promising multifunctional photoprotective systems, combining UV attenuation with antioxidant, anti-inflammatory, and biologically adaptive properties. Plant extracts are increasingly used as reducing and stabilising agents in the green synthesis of metal and metal oxide nanoparticles. Among these, ZnO and TiO_2_ serve as established inorganic UV filters, whereas Ag and Au nanoparticles have primarily been investigated for their antioxidant, anti-inflammatory, antimicrobial, and ROS-modulating properties, which may indirectly enhance photoprotection. In parallel, plant-derived organic nanoparticles and herbal nanocomposites have demonstrated enhanced biocompatibility and multifunctional performance. This review critically examines the current landscape of plant-mediated photoprotective nanomaterials, focusing on the mechanistic interplay among optical UV attenuation, reactive oxygen species (ROS) modulation, and cellular signalling regulation. Particular emphasis is placed on structure–function relationships governing nanoparticle size, surface chemistry, bandgap properties, antioxidant behaviour, and biological interactions. The review further discusses translational challenges, including reproducibility, standardisation, scalability, long-term safety, regulatory classification, and limitations in benchmarking. Importantly, current evidence suggests that no single material system simultaneously optimises UV-blocking efficiency, ROS control, biocompatibility, and industrial scalability, highlighting the need for multifunctional hybrid design strategies. Finally, future perspectives involving predictive nanoengineering, computational modelling, machine learning-guided optimisation, and adaptive photoprotective systems are discussed as emerging directions for next-generation sustainable photoprotective technologies.

## 1. Introduction

Ultraviolet (UV) radiation is a major environmental stressor that contributes to a wide spectrum of acute and chronic skin disorders [1,2]. Excessive exposure to UVA and UVB radiation induces oxidative stress, inflammation, erythema, DNA damage, immunosuppression, extracellular matrix degradation, and skin barrier dysfunction, ultimately contributing to premature skin ageing (photoaging) and photocarcinogenesis [3,4,5]. Consequently, effective photoprotection is essential not only for reducing the risk of skin cancer but also for preserving skin integrity, preventing premature ageing, and maintaining long-term skin health. Chronic UV exposure accelerates photoaging through ROS-mediated activation of NF-κB, AP-1 and MAPK signalling, resulting in inflammation, extracellular matrix degradation and impaired skin-barrier function [3,4].

Nanotechnology addresses key limitations of plant-derived photoprotective compounds, including poor aqueous solubility, photoinstability, rapid degradation, and limited skin retention, by enhancing stability, enabling controlled delivery, and improving local bioavailability [6,7]. Many phytochemicals, including flavonoids, polyphenols, carotenoids, and phenolic acids, exhibit potent antioxidant and anti-inflammatory activities that help protect the skin against UV-induced oxidative stress, DNA damage, and inflammatory responses [4,5,8]. However, their practical application is often limited by poor aqueous solubility, low photostability, and limited skin penetration [7,9]. Green nanotechnology addresses these limitations by employing plant extracts as reducing and stabilising agents for the environmentally friendly synthesis of nanoparticles. Importantly, the phytochemicals present in these extracts, including phenolics, flavonoids, terpenoids, and proteins—not only drive nanoparticle formation but frequently remain associated with the nanoparticle surface as capping agents. These surface-bound phytochemicals contribute directly to photoprotection by scavenging UV-induced reactive oxygen species, suppressing inflammatory signalling, improving colloidal stability, and, in some systems, reducing photocatalytic ROS generation. Consequently, plant-mediated nanomaterials derive their photoprotective performance from the combined effects of the inorganic nanoparticle core, which primarily attenuates UV radiation, and the biologically active plant-derived surface components, which mitigate oxidative and inflammatory damage [9,10,11]. Plant-mediated nanoparticles have demonstrated considerable potential for improving photoprotection by enhancing the stability, bioavailability, and skin retention of photoprotective phytochemicals [6,12]. These improvements arise because nanoscale carriers increase the interfacial contact between phytochemicals and the skin surface, protect bioactive compounds from UV-induced degradation, and enable sustained release, thereby prolonging antioxidant and anti-inflammatory activity. Nevertheless, plant-mediated synthesis should not be interpreted as an inherent guarantee of safety. The biological behaviour of nanomaterials depends on multiple factors, including particle composition, size, surface chemistry, formulation, dosage, and exposure conditions [13,14,15]. Therefore, comprehensive toxicological evaluation remains essential before their widespread cosmetic or clinical application [13,16].

**Figure 1:**
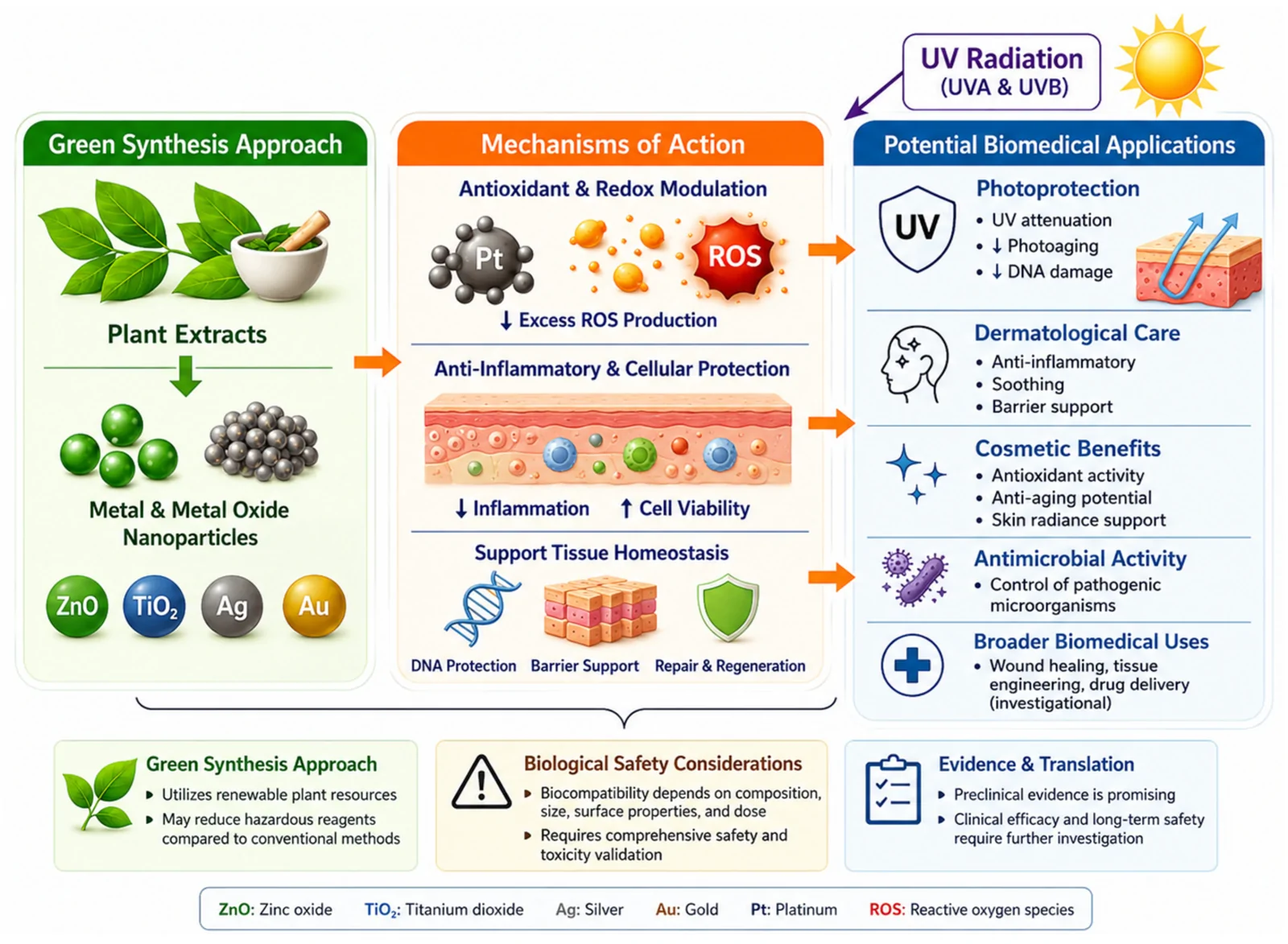
Schematic representation of the complementary photoprotective mechanisms of plant-mediated nanomaterials. The inorganic nanoparticle core attenuates UV radiation through absorption and scattering, while plant-derived surface phytochemicals provide antioxidant, ROS-scavenging, and anti-inflammatory functions, together reducing UV-induced oxidative stress and skin damage.

Importantly, ZnO and TiO_2_ are established direct inorganic UV filters, whereas Ag and Au nanoparticles contribute primarily through indirect antioxidant and anti-inflammatory mechanisms (Figure 1). In cosmetics, ZnO nanostructures are commonly incorporated into sunscreens as inorganic filters that attenuate UVA and UVB radiation. Reinosa et al. developed a hierarchical composite comprising ZnO nanoparticles anchored onto TiO_2_ microparticles, which exhibited greater UV absorption than pure ZnO or TiO_2_ and an approximately 18% higher initial SPF than TiO_2_ microparticles alone [17].

To provide a structured understanding of this rapidly evolving field, plant-mediated photoprotective nanomaterials can be categorised based on their dominant mechanism and functional design [7,13]. These include: (i) inorganic UV-blocking nanoparticles (e.g., ZnO, TiO_2_), which primarily act through photon absorption and scattering; (ii) phytochemical-based systems, which mitigate UV-induced damage via antioxidant and ROS-scavenging activity; (iii) hybrid nanocomposites that integrate inorganic and organic components to achieve synergistic photoprotection; and (iv) emerging smart nanomaterials designed for controlled or stimuli-responsive release of protective agents [6,7,18,19]. In this review, we propose a unified design framework in which photoprotective performance emerges from the interplay between (i) optical UV attenuation, (ii) redox modulation of reactive oxygen species, and (iii) regulation of cellular signalling pathways. This framework provides a basis for the rational design of next-generation photoprotective nanomaterials [7,13].

Despite rapid progress, the field remains largely descriptive, with limited integration between nanoparticle physicochemistry, ROS behaviour, and biological responses [6,13]. The absence of predictive structure–function relationships currently limits the rational engineering and clinical translation of plant-mediated photoprotective nanomaterials. Most studies remain descriptive, focusing on synthesis and isolated functional outcomes without systematically defining structure–function relationships or addressing the trade-offs between UV attenuation, reactive oxygen species (ROS) generation, and biocompatibility [6,7,16]. This limits the rational design and clinical translation of plant- mediated photoprotective nanomaterials. In this review, the term plant-derived nanomaterials refers broadly to nanomaterials produced using plant-derived components. These include plant-mediated inorganic nanoparticles, plant-derived organic nanoparticles, herbal nanocomposites, and phytochemical-loaded nanocarriers. Although these systems share a botanical origin, they differ in their composition, synthesis methods, and mechanisms of photoprotection.

To provide a logical framework, this review is organised around the major determinants of photoprotective performance. We first discuss plant-derived photoprotective compounds and the major classes of plant-mediated nanomaterials, and then examine advances in green nanoparticle synthesis and hybrid bionanocomposites. We then examine the physicochemical factors governing UV attenuation, ROS modulation, and biological interactions before discussing safety, translational challenges, and future perspectives. This structure links material design with biological function and clinical translation.

## 2. Materials

### Literature Search Strategy

A structured literature search was conducted in the Web of Science, Scopus, and PubMed databases to identify studies on plant-mediated nanomaterials for photoprotective applications. The search strategy combined keywords and Boolean operators, including: “plant-mediated nanoparticles”, “green synthesis”, “biosynthesized nanoparticles”, “phytochemical-based nanomaterials”, “bionanocomposites”, “UV protection”, “photoprotection”, “UV absorption”, “photoaging”, “photocarcinogenesis”, and “reactive oxygen species”.

Studies published between 2005 and 2025 were considered, with particular emphasis on publications from 2018 onward to capture recent advances in nanoparticle synthesis, photoprotective mechanisms, safety evaluation, and translational applications. Original research articles, systematic reviews, and high-quality review papers published in peer-reviewed journals were included.

Studies were selected based on relevance to photoprotective nanomaterials derived from plant extracts or plant-based biomaterials. Priority was given to studies reporting mechanistic insights, physicochemical characterization, ROS modulation, UV-blocking performance, biological evaluation, or translational relevance. Non-English publications, conference abstracts, duplicated reports, and studies lacking sufficient methodological detail were excluded. Although this review is narrative rather than systematic, efforts were made to critically evaluate the consistency, mechanistic depth, and translational significance of the available evidence.

## 3. Results

### 3.1. Herbal Products

Natural phytochemicals have long attracted attention for photoprotective skincare applications because many plant-derived compounds possess antioxidant, anti-inflammatory, and UV-absorbing properties. Polyphenols, flavonoids, terpenoids, lignins, and phenolic acids can reduce oxidative stress and mitigate UV-induced cellular damage through ROS scavenging and modulation of signalling [20,21]. Compounds such as quercetin, rutin, curcumin, catechins, and silymarin have demonstrated protective effects against photoaging, inflammation, and photocarcinogenesis in experimental models [8,22]. Growing consumer demand for naturally derived skincare products has accelerated interest in integrating phytochemicals into nanoscale systems [9].

Compared with free compounds, nanoscale delivery systems may improve stability, bioavailability, skin retention, and sustained antioxidant activity [10,12]. However, despite promising biological activity, direct comparisons across studies remain difficult due to variability in extraction methods, phytochemical composition, UV exposure conditions, and experimental models. Consequently, the relative contribution of intrinsic phytochemical activity versus that of the nanoscale formulation remains incompletely understood [23].

For clarity, the terminology used throughout this review is based on the composition and synthesis strategy of the nanomaterials. Plant-mediated nanoparticles are inorganic nanoparticles (e.g., ZnO and TiO_2_) synthesised using plant extracts as reducing and stabilising agents via green synthesis. In contrast, plant-derived organic nanoparticles are fabricated directly from plant-derived materials, such as lignin, cellulose, or other phytopolymers. Herbal nanocomposites comprise hybrid systems that combine inorganic nanoparticles with plant-derived polymers or phytochemicals, whereas phytochemical-loaded nanocarriers encapsulate plant bioactive compounds (e.g., curcumin or resveratrol) to improve their stability, bioavailability, and photoprotective efficacy. These definitions are used consistently throughout this review. While free phytochemicals possess antioxidant and anti-inflammatory activity, their limited stability and bioavailability have driven the development of nanomaterial-based delivery systems. Accordingly, the following section categorises the principal classes of plant-derived photoprotective nanomaterials.

### 3.2. Categories of Plant-Derived Nanomaterials in Photoprotective Applications

Based on these definitions, plant-derived nanomaterials used for UV protection can be broadly categorised according to their composition and synthesis method [6,12,24]. Understanding these categories clarifies the diverse strategies used to develop eco-friendly photoprotective systems [7,13]. The first category comprises plant-mediated inorganic nanoparticles, in which plant extracts act as reducing and stabilising agents during the green synthesis of metal or metal oxide nanoparticles [9,10,12]. In this approach, plant extracts function not only as reducing and stabilising agents during nanoparticle synthesis but also as sources of bioactive phytochemicals that frequently remain adsorbed on the nanoparticle surface [9,11]. These surface-associated flavonoids, phenolics, terpenoids, and proteins influence nanoparticle surface chemistry and contribute directly to photoprotection through antioxidant, ROS-scavenging, and anti-inflammatory activities [10]. Consequently, plant-mediated nanoparticles derive their functionality from both the inorganic nanoparticle core and the biologically active phytochemical corona [12,25,26,27].

An alternative category is plant-derived organic nanoparticles, produced directly from plant-based polymers such as lignin, cellulose, and other photopolymers [18,28,29]. While these materials possess intrinsic antioxidant properties, their photoprotective performance remains inconsistent due to compositional variability and limited standardisation across studies [18,28]. A third category comprises herbal nanocomposites, which combine inorganic nanoparticles with plant-derived polymers or phytochemicals to achieve complementary photoprotective functions. Hybrid nanocomposites frequently outperform single-component systems because they integrate complementary mechanisms, whereby inorganic components (e.g., ZnO and TiO_2_) provide efficient UV attenuation, while phytochemical-rich organic phases contribute antioxidant, anti-inflammatory, and ROS-scavenging activities, thereby enhancing both photoprotective performance and biological compatibility [7,13,18,24]. This synergistic behaviour may enhance photoprotective performance and biological compatibility by integrating complementary mechanisms of UV attenuation and antioxidant defence [6,18,19]. In these systems, plant-derived antioxidants, such as curcumin, catechins, and resveratrol, are encapsulated in nanocarriers to enhance their physicochemical stability, bioavailability, and sustained photoprotective activity [7,30,31,32].

While these categories demonstrate the versatility of plant-derived nanomaterials, their comparative performance remains poorly defined [7,13,16]. Few studies systematically evaluate differences in UV-blocking efficiency, ROS modulation, and long-term stability across material types under comparable conditions. As a result, the field lacks clear structure–function relationships that link composition and synthesis strategy to photoprotective performance [6,13,15]. Consequently, direct comparisons across material systems remain difficult, limiting the identification of optimal design parameters for translational photoprotective applications [15,16].

### 3.3. Advances and Limitations in Plant-Based Nanoparticle Technology

The technology for synthesising metal nanoparticles using plant extracts has advanced significantly [10,11,33]. Meanwhile, the production and application of plant nanoparticles are still in their early stages [15,16]. Due to their high safety and non-toxicity, plant nanoparticles have provided a promising and growing platform for advances in pharmaceutical sciences. For example, in 2017, a study investigated and demonstrated the antibacterial and larvicidal properties of Acalypha indica nanoparticles [34]. In addition, a study by Subramani et al. demonstrates the UV-blocking and antimicrobial properties of herbal nanoparticles prepared from *Aloe vera* leaves [35]. Likewise, Kumar’s 2018 research demonstrated that the UV absorption spectra of all herbal nanoparticles from selected mangrove plants exhibited absorption ranging from 223 nm to 664 nm in *Avicennia marina*, *Rhizophora apiculata*, and *Excoecaria agallocha*, respectively [36]. Although plant-derived nanoparticles show significant promise, the field remains in its early stages, with limited standardisation, insufficient mechanistic evaluation, and relatively few studies validating their performance in physiologically relevant or in vivo models. Given the high efficacy of medicinal plants in medicine and pharmaceutical sciences, these nanoparticles have strong potential to advance nanotechnology in these fields. Therefore, plant nanoparticles can be introduced as a suitable path for further studies.

Despite these promising findings, the development of plant-derived nanoparticles remains limited by several challenges [10,12,16]. Variability in plant extract composition can lead to poor reproducibility and inconsistent nanoparticle properties across studies. In addition, most reports rely heavily on in vitro assays, with limited validation in physiologically relevant or in vivo models [15,16,33]. These limitations highlight the need for standardised synthesis protocols and more rigorous biological evaluation to enable clinical translation [13,15,16]. Although numerous plant-derived nanoparticles have been reported, their performance varies significantly depending on plant source, extraction method, and synthesis conditions [10,12]. For example, nanoparticles derived from polyphenol-rich extracts such as green tea or pomegranate generally exhibit stronger antioxidant and ROS-scavenging activity, whereas structurally derived plant nanoparticles (e.g., lignin or cellulose-based systems) tend to provide greater stability but comparatively weaker bioactivity [18,28,32,37]. However, most studies do not standardise experimental conditions, making direct comparison difficult. This lack of consistency limits the identification of optimal design parameters and highlights the need for systematic benchmarking across material systems [6,13,16].

### 3.4. Plant-Mediated Synthesis of Nanoparticles

Phytochemicals such as polyphenols, flavonoids, terpenoids, alkaloids, proteins, and polysaccharides facilitate nanoparticle formation and regulate nucleation, crystal growth, and stabilization. While these biomolecules reduce metal ions during the synthesis of metallic nanoparticles (e.g., Ag and Au), plant-mediated ZnO nanoparticles are formed via hydrolysis and condensation reactions, with phytochemicals primarily acting as capping and growth-regulating agents [12,33,38,39,40]. The resulting nanoparticle surface is typically coated with both unmodified phytochemicals and their oxidation products, which together act as capping agents and regulate crystal growth, colloidal stability, surface chemistry, and subsequent biological interaction [12,33,38,39,40,41]. As a result, the composition of the phytochemical corona influences nanoparticle stability and biological functions, including antioxidant, anti-inflammatory, and ROS-scavenging activities [4,5,24,41]. The properties and biological effects of plant-based nanomaterials can be tailored by adjusting synthesis parameters like plant species, extract composition, precursor concentration, temperature, reaction time, and surface functionalization [12,33,39,40,41]. These factors determine particle size, shape, crystallinity, surface charge, and phytochemical loading, impacting UV attenuation, photocatalytic activity, ROS production, and overall photoprotective performance [5,7,40].

During the synthesis of metallic nanoparticles, many phytochemicals are not merely adsorbed onto the nanoparticle surface but are chemically transformed through oxidation, donating electrons to reduce metal ions [38,42]. Polyphenols and flavonoids, for example, are commonly oxidised to quinone or semiquinone derivatives, resulting in changes to their hydroxyl, carbonyl, and conjugated functional groups [43,44]. These oxidation products differ from the parent compounds in their redox activity, metal-binding affinity, antioxidant capacity, and surface interactions [43,44,45]. Consequently, the phytochemical corona surrounding plant-mediated nanoparticles is often a complex mixture of native and oxidised biomolecules that collectively influence nanoparticle stability, surface chemistry, biological interactions, and photoprotective performance [42,46,47]. In contrast, during the synthesis of metal oxide nanoparticles such as ZnO, phytochemicals primarily regulate hydrolysis, nucleation, crystal growth, and surface stabilization, with comparatively less direct oxidation associated with metal-ion reduction [42,48,49].

**Figure 2:**
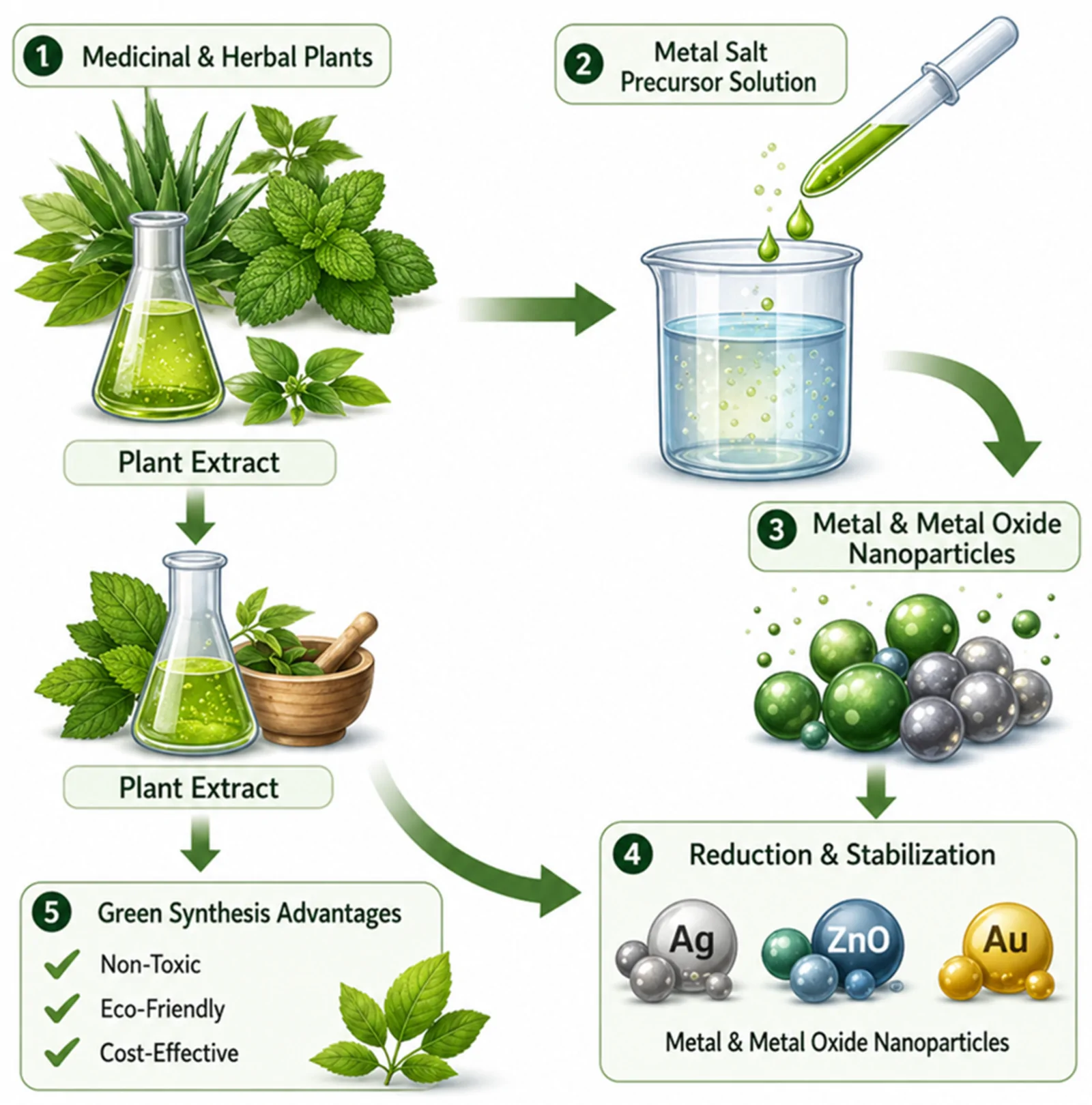
Green synthesis of metal and metal oxide nanoparticles using medicinal and herbal plant extracts.

Plant-mediated green synthesis provides an environmentally friendly strategy for producing metal and metal oxide nanoparticles using bioactive phytochemicals as natural reducing and stabilising agents (Figure 2) [11]. Biological methods using microorganisms and plant products offer several advantages over physical and chemical methods, including cost-effectiveness, eco-friendliness, and scalability [50]. Natural biogenic metallic nanoparticles can be synthesised via various biological mechanisms. One major category is bioreduction, in which microorganisms and their enzymes reduce metal ions into stable metallic nanostructures. Another category is biosorption, in which metal cations bind to the cell walls of organisms in aqueous media, forming stable nanoparticles [51,52,53]. Nanoparticle synthesis using bacteria offers several advantages, including their abundant availability, adaptability to extreme environmental conditions, rapid multiplication, and ease of cultivation and manipulation. However, this approach also has certain disadvantages, particularly potential safety risks associated with handling and using bacterial systems [51].

Importantly, plant-mediated synthesis is not merely an environmentally friendly alternative to conventional nanoparticle production; it also fundamentally alters nanoparticle surface chemistry and biological behaviour. Surface-bound phytochemicals not only improve colloidal stability but also actively participate in photoprotection by scavenging UV-induced reactive oxygen species, reducing inflammatory signalling, limiting photocatalytic ROS formation, and enhancing biological compatibility. Consequently, the plant-derived surface layer should be regarded as a functional component of the nanomaterial rather than simply a synthesis residue [9,10,11,15,33,54].

A study by Yoshihisa and colleagues highlights the protective effects of platinum nanoparticles (nano-Pt) against UV-induced skin damage [55]. The study found that UV exposure increases ROS production in HaCaT keratinocytes, whereas nano-Pt treatment significantly reduces ROS levels. Additionally, pretreatment with nano-Pt markedly inhibits UVB- and UVC-induced apoptosis in keratinocytes. In vivo experiments further demonstrated that mice treated with nano-Pt gel prior to UV exposure exhibited reduced UVB-induced inflammation and decreased UVA-induced photoallergic responses compared with untreated controls. Overall, these findings suggest that nano-Pt mitigates UV-induced skin damage by reducing ROS production and preventing cell apoptosis.

*Zingiber officinale* extract can act as a reducing agent and stabiliser for metallic nanoparticles with diameters ranging from 5 to 15 nm. Factors affecting nanoparticle synthesis include extract concentration, metal salt concentration, temperature, and pH. Nanoparticles with controlled size and morphology can be produced using plant extracts, such as those from *Polyalthia longifolia*. Nanoparticles are used in sunscreen lotions for UV protection [56].

In another study, multiphase crystalline Zn-Aloe vera nanostructures were synthesised using visible light photocatalysis, exhibiting UV to visible light activity with a bandgap of 3.37 eV to 2.5 eV. Aloe Vera enhanced the material’s visible-light photocatalytic properties, making it promising for biomedical applications, including anti-cancer, antibacterial, and anti-diabetic effects, as well as photo-degradation [57]. ZnO and TiO_2_ are low-cost, eco-friendly, non-toxic metal oxides used for UV protection in sunscreens and coatings. They absorb UV radiation and convert it into harmless infrared light while maintaining high transparency in the visible spectrum [18].

Plant extracts containing bioactive phytochemicals act as reducing agents during the synthesis of metallic nanoparticles (e.g., Ag and Au) and as capping, stabilising, and growth-regulating agents during the synthesis of metal oxide nanoparticles (e.g., ZnO and TiO_2_). For ZnO, nanoparticle formation occurs primarily through hydrolysis and condensation of zinc precursors, while phytochemicals regulate nucleation, crystal growth, and surface functionalisation [9,10,11]. When mixed with metal salt precursor solutions, these compounds reduce metal ions to form nanoparticles such as ZnO, Ag, and Au, while simultaneously adsorbing onto the nanoparticle surface as stabilising capping agents [9,10,11,33]. This green synthesis approach provides a sustainable, cost-effective, and environmentally friendly alternative to conventional nanoparticle production methods [10,15,16]. However, the chemical complexity and variability of plant extracts present significant challenges to reproducibility and mechanistic understanding, as the specific biomolecules responsible for nanoparticle reduction, stabilisation, and surface functionalisation are often poorly defined. Furthermore, scale-up remains challenging due to variability in raw materials and sensitivity to reaction conditions. Addressing these limitations through standardised extraction protocols, phytochemical profiling, improved process control, rigorous physicochemical characterisation, and quality-control frameworks will be critical for improving reproducibility and enabling translation from laboratory-scale synthesis to industrial manufacturing [10,15,16].

### 3.5. Design Rules for Plant-Derived Photoprotective Nanomaterials

The preceding sections demonstrate that photoprotective efficacy is determined not by a single material property but by the interplay between optical behaviour, antioxidant capacity, and biological interactions. These observations can be consolidated into a series of design principles that guide the rational engineering of next-generation photoprotective nanomaterials. The development of effective photoprotective nanomaterials requires careful balancing of competing physicochemical and biological parameters. Current evidence suggests several key design principles governing performance and safety.

Particle size plays a critical role: smaller nanoparticles enhance UV scattering efficiency but also increase the risk of skin penetration, with an optimal size range generally considered to be approximately 50–150 nm [14,58]. Bandgap engineering is equally important, as it determines the spectral range of UV absorption; for example, tuning materials such as ZnO and TiO_2_ is essential for targeting UVA versus UVB protection [59]. Surface chemistry further modulates functionality, with inert coatings such as silica or polymers reducing the generation of reactive oxygen species (ROS), while phytochemical capping imparts intrinsic antioxidant properties [14]. Importantly, ROS itself has a dual role, contributing beneficial antimicrobial effects while also posing risks of oxidative stress, necessitating precise control over its generation and scavenging [60].

Composite design offers another critical advantage, as hybrid systems often outperform single-component materials; for instance, lignin–ZnO nanocomposites provide synergistic UV-blocking and antioxidant effects [61]. Finally, the biological interface must be carefully engineered to minimise adverse responses, including inflammation and immune activation, with surface functionalisation playing a key role in modulating these interactions. Collectively, these principles demonstrate that photoprotective performance is not determined by a single parameter but rather emerges from the complex interplay among optical properties, redox behaviour, and biological responses.

### 3.6. Bionanocomposites

Hybrid bionanocomposites integrate inorganic nanoparticles with plant-derived polymers or phytochemicals to combine complementary optical, antioxidant, and biological functions. In these systems, the inorganic phase primarily provides broadband UV attenuation through photon absorption and scattering, whereas the organic phase suppresses oxidative stress by scavenging ROS, limiting inflammatory signalling, and improving colloidal stability [62,63,64].

Lignin possesses intrinsic UV-absorbing properties because its aromatic phenolic network absorbs UV photons, while abundant phenolic hydroxyl groups donate hydrogen atoms to neutralise free radicals generated during UV exposure [18,28,61]. Similarly, chitosan-based nanocomposites can enhance colloidal stability, film formation, controlled release behaviour, and biological compatibility [29,65]. Hybrid systems may also reduce excessive photocatalytic ROS generation by modulating electron–hole recombination at nanoparticle interfaces [28].

Despite these advantages, systematic benchmarking across different bionanocomposite systems remains limited. Most studies use different synthesis conditions, UV exposure models, particle sizes, and biological assays, making direct comparison difficult. Establishing standardised evaluation protocols will therefore be essential for identifying optimal hybrid photoprotective systems [13,18,28,66].

Plasma-driven surface functionalization approaches have demonstrated precise control over nano-bio interfaces, significantly influencing cellular compatibility and inflammatory responses, both of which are critical for the safe development of advanced bionanocomposites [66]. Further research in this area is needed to expand the applicability of these sustainable materials across various fields. Key differences between conventional UV filters and emerging plant-derived nanomaterials are summarised in Table 1.

Collectively, these comparisons demonstrate that no single material system currently optimises UV attenuation, ROS regulation, biocompatibility, scalability, and regulatory readiness simultaneously, highlighting the need for multifunctional hybrid design strategies.

### 3.7. Interaction of UV Radiation with Nanomaterials

At the nanoscale, photoprotective performance is strongly influenced by particle size, morphology, crystallinity, bandgap energy, and surface chemistry [6,13,14,59]. Smaller nanoparticles generally exhibit increased UV scattering and surface reactivity due to their high surface area-to-volume ratio [6,59]. However, excessive particle-size reduction may also increase photocatalytic ROS generation and raise concerns about skin penetration and biological interactions [13,14,68]. Consequently, photoprotective efficacy and nanosafety are intrinsically interconnected design parameters rather than independent considerations. Nanoparticle aggregation also plays an important role, as agglomeration decreases the effective surface area, alters light scattering behaviour, reduces colloidal stability, and can modify photocatalytic activity and ROS generation. Consequently, controlling particle dispersion is critical for achieving reproducible UV attenuation and biological performance [6,13,54].

UV radiation, with its high energy and short wavelength, can induce unique photophysical and photochemical effects when it interacts with nanoscale materials. These interactions can lead to enhanced optical properties, improved photocatalytic activity, and novel functionalities not observed in bulk materials [6,13,69]. Understanding these interactions is crucial for advancing applications in areas such as photovoltaics, environmental remediation, biomedical devices, and advanced manufacturing. Nanomaterials, particularly ZnO and titanium dioxide (TiO_2_) nanoparticles, are commonly used in sunscreens due to their superior ability to block or absorb UV radiation. These nanoparticles typically range in size from 1 to 100 nanometers. They scatter and absorb UV radiation more effectively than larger particles due to their small size and high surface area-to-volume ratio. TiO_2_ nanoparticles primarily absorb UVB radiation, whereas ZnO nanoparticles provide broad-spectrum protection across both the UVA and UVB regions [14,59,69]. When UV photon energy exceeds the bandgap of ZnO or TiO_2_, electrons are excited from the valence band to the conduction band, leaving electron–hole pairs. These charge carriers may recombine or react with oxygen and water to generate ROS. The surrounding microenvironment, including pH, oxygen availability, aggregation, antioxidants and surface coatings, regulates these reactions and therefore influences photoprotective efficacy and biological safety [48,70,71].

For plant-mediated nanomaterials, photoprotection is achieved through two complementary mechanisms. The inorganic nanoparticle core attenuates UV radiation through absorption and scattering [14,17,18,59,69], while plant-derived surface phytochemicals reduce UV-induced oxidative stress by scavenging reactive oxygen species and modulating inflammatory signalling [4,5,7,19,72]. In addition, these phytochemicals may suppress excessive photocatalytic activity by influencing charge-carrier dynamics, recombination and surface redox reactions, thereby improving both photoprotective efficacy and biological safety [18,22,29].

To mitigate potential photocatalytic damage, nanoparticles are often coated with inert materials such as silica or alumina. These coatings help stabilise the particles, reducing ROS generation while maintaining their UV-blocking properties [73]. In addition, nanoengineered surface modifications have been reported to significantly influence ROS production and subsequent immune cell responses, highlighting the critical role of nanoscale interface design in improving the biological safety of functional nanomaterials [66].

A previous study demonstrated that the antagonistic interaction observed in genotoxic effects is linked to exposure to ZnO nanoparticles and UV light. Interestingly, this interaction was noted across all biomarkers used: comet assay, micronuclei formation, and anchorage-independent growth. These effects do not appear to be related to increased toxicity from the combined treatments. The use of such nanoparticles in cosmetics and sunscreens has sparked debate over whether they can penetrate the skin, enter the bloodstream, and spread throughout the body. However, based on current information, their potential risk through topical exposure is considered very low [68]. Metal deposition on TiO_2_ can reduce photocatalytic activity by promoting charge-carrier recombination at the metal–TiO_2_ interface, demonstrating how composition and interfacial structure regulate UV-induced ROS generation [74,75].

**Figure 3:**
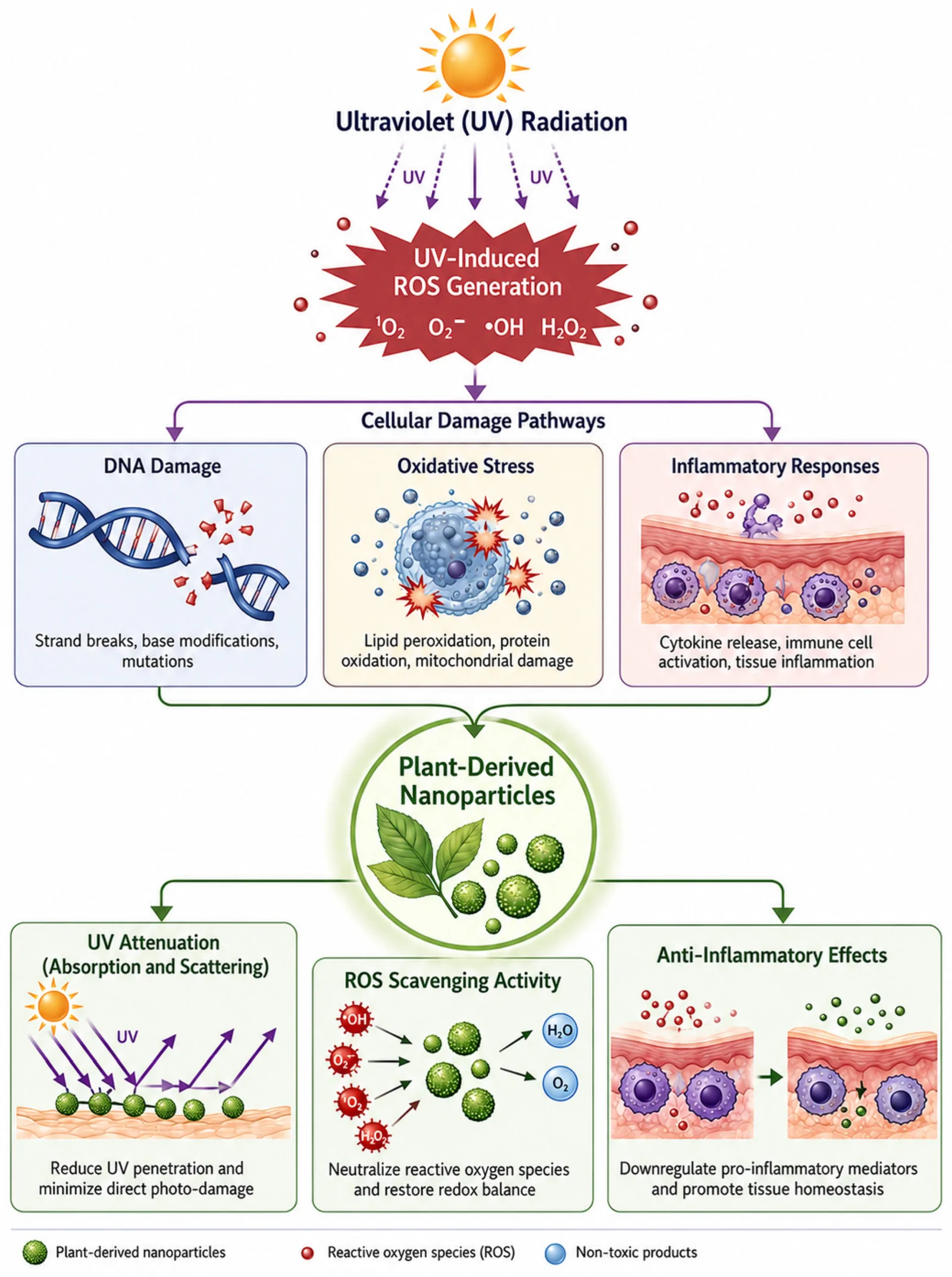
Mechanisms of photoprotection by plant-derived nanomaterials. UV radiation induces ROS, leading to oxidative stress, DNA damage, and inflammation. Plant-derived nanoparticles provide protection by absorbing UV radiation, scavenging ROS, and reducing inflammatory responses.

Beyond direct UV attenuation, modulation of oxidative stress represents a central mechanism underlying photoprotection. UV radiation induces ROS, which activate signalling pathways such as NF-κB, AP-1, and MAPK, leading to oxidative stress, inflammation, and extracellular matrix degradation (Figure 3) [4,5,6,7]. Phytochemicals such as flavonoids and polyphenols act as potent antioxidants, neutralising ROS and mitigating UV-induced cellular damage [7]. Mechanistically, polyphenols and flavonoids donate electrons through hydroxyl functional groups to reduce Ag^+^ and Au^3+^ ions, whereas proteins, polysaccharides, and terpenoids preferentially adsorb to growing crystal surfaces to regulate nucleation, crystal growth, aggregation, and surface chemistry. During ZnO synthesis, hydrolysis and condensation of zinc precursors generate Zn(OH)_2_ intermediates that subsequently dehydrate to ZnO, while phytochemicals primarily regulate crystal growth rather than directly reducing metal ions [12,38,76]. Plant-derived nanoparticles, including platinum nanoparticles, ginseng-derived exosome-like nanoparticles and phytochemical-capped ZnO nanomaterials, have been shown to reduce ROS production, suppress NF-κB/AP-1/MAPK signalling, decrease inflammatory cytokine production and inhibit apoptosis in UV-exposed keratinocytes and experimental animal models [72]. In addition, many plant-derived compounds exert anti-inflammatory effects. These bioactive molecules can inhibit inflammatory signalling pathways such as NF-κB and MAPK, which are typically activated following UV exposure. By suppressing these pathways, the production of pro-inflammatory cytokines is reduced, thereby improving skin protection [4,5,7,72].

Collectively, these interconnected mechanisms reduce UV-induced oxidative stress, inflammation, DNA damage, and apoptosis [4,5,6,72]. Importantly, their relative contributions depend strongly on nanoparticle size, composition, bandgap properties, and surface chemistry, underscoring the need for integrated structure–function design strategies [6,13,14]. By limiting oxidative stress and inflammation, plant-derived nanomaterials help prevent UV-induced DNA damage and apoptosis in skin cells. Such protective effects are crucial for reducing long-term consequences of UV exposure, including photoaging and photocarcinogenesis [4,7,72]. Ongoing research into safety profiles ensures that these benefits do not come at an undue risk to human health or the environment, paving the way for safer sun protection solutions in future skincare products [13,16,77].

Representative studies demonstrate that the biological activity of plant-mediated nanomaterials depends strongly on nanoparticle composition and experimental conditions [6,7,13]. Platinum nanoparticles reduced UVB- and UVC-induced ROS production and apoptosis in HaCaT keratinocytes and attenuated UV-induced skin inflammation in mice by suppressing oxidative stress [55]. Similarly, ginseng-derived exosome-like nanoparticles protected UV-exposed skin by limiting ROS generation and inhibiting AP-1 signalling, while phytochemical-rich ZnO nanomaterials reduced oxidative stress and inflammatory responses through antioxidant activity [25,57,72]. Collectively, current evidence indicates that modulation of NF-κB, AP-1, MAPK signalling, apoptosis, and inflammatory cytokine production is primarily mediated by ROS regulation rather than by direct pathway-specific targeting [4,5,6,7,72]. However, comparisons between studies remain difficult because UV wavelengths, radiation doses, nanoparticle characteristics, and biological models vary considerably [7,13,77].

### 3.8. Practical Implementation of Nanoparticles in Photobioprotection

ZnO and TiO_2_ nanoparticles are incorporated into sunscreens and cosmetics because they efficiently absorb and scatter UV radiation while maintaining greater transparency than larger particles. ZnO provides broad UVA and UVB protection, whereas TiO_2_ primarily attenuates UVB radiation. Surface coatings are commonly applied to improve dispersion and reduce photocatalytic ROS generation [14,59,78].

Commercial examples of nanoparticulate photoprotective materials include Eusolex^®^ T-PURE and Eusolex^®^ T-2000, which contain surface-treated nano-TiO_2_; Eusolex^®^ T-AVO, a silica-coated nano-TiO_2_ filter; and Eusolex^®^ Z-TEC, which contains nano-ZnO (Merck KGaA, Darmstadt, Germany) [79]. Other examples include Croda’s Solaveil™ CT-300 and Solaveil™ CZ-300 (Croda International Plc, Snaith, UK), transparent mineral UV-filter grades based on TiO_2_ and ZnO, respectively [80]. These branded raw materials are incorporated into sunscreen, skincare and cosmetic formulations to provide UV attenuation while reducing the visible whitening commonly associated with larger mineral particles. Product availability, particle specifications and regulatory classification may vary between jurisdictions and formulations.

Beyond sunscreens, these nanoparticles are incorporated into textiles, transparent polymer coatings and protective films to reduce UV transmission and material degradation. Hybrid systems, including lignin–ZnO and silica–ZnO composites, can additionally provide antioxidant, antibacterial and self-cleaning properties [20,21,29,61,81,82]. Practical application requires careful control of nanoparticle concentration, aggregation, coating stability and potential environmental release.

### 3.9. Safety and Toxicology

Although plant-mediated nanomaterials are often described as biocompatible or environmentally friendly, their long-term biological safety remains incompletely understood. Safety profiles depend strongly on nanoparticle composition, size, crystallinity, aggregation behaviour, surface chemistry, dosage, exposure duration, and formulation conditions [74,75]. Furthermore, the biological response reported in the literature varies substantially depending on the experimental model employed. Most mechanistic studies have been performed in keratinocyte cell lines (e.g., HaCaT), fibroblasts or reconstructed skin models following UVA or UVB irradiation, whereas relatively few studies have validated these findings in animal models. This heterogeneity limits direct comparison of signalling pathways and biological outcomes across studies [4,7,77].

One of the primary concerns involves nanoparticle penetration through compromised or damaged skin barriers. While most evidence suggests that ZnO and TiO_2_ nanoparticles largely remain within the stratum corneum under normal conditions, penetration behaviour may vary depending on particle size, coating chemistry, aggregation state, UV exposure, and skin integrity. Chronic exposure conditions remain insufficiently characterised [74,75].

Another major concern relates to photocatalytic ROS generation. ROS generation is governed by crystal phase, surface defects, oxygen vacancies, particle size, and electron-hole recombination kinetics [48,71]. Surface coatings such as silica, alumina, polymers, or phytochemical capping reduce charge-carrier transfer to oxygen and water, thereby limiting photocatalytic ROS production without substantially attenuating UV [14,59,70].

The biological effects of ROS are dose-dependent. Low or transient ROS levels may be beneficial for antimicrobial activity, whereas excessive or prolonged ROS production can damage healthy cells and promote oxidative stress. Safety assessments should therefore consider both the magnitude and duration of ROS generation [83,84].

Environmental accumulation and ecotoxicological effects also represent emerging challenges. Nanoparticles released from sunscreen formulations may interact with aquatic ecosystems and biological organisms, raising concerns regarding environmental persistence and bioaccumulation [1,13]. Consequently, comprehensive in vitro, in vivo, and environmental safety evaluation will be essential before widespread clinical and commercial implementation [13,16]. Importantly, the term “green” should not be interpreted as inherently non-toxic. Plant-mediated synthesis may reduce hazardous chemical exposure during production, but the resulting nanomaterials still require rigorous toxicological evaluation using standardised biological models and long-term exposure studies [15,16]. Although plant-mediated synthesis reduces the use of hazardous chemicals during nanoparticle production, the resulting nanomaterials may still induce adverse biological responses depending on their composition, size, surface chemistry, dose, and exposure conditions. Therefore, each material requires comprehensive safety evaluation rather than being assumed to be biocompatible because of its green synthesis route.

### 3.10. Environmental Toxicity and Biodistribution

The environmental effects of ZnO and TiO_2_ nanoparticles vary substantially with concentration, particle size, aggregation, exposure duration, biological model and soil properties. In agricultural soil, 21 nm TiO_2_ nanoparticles applied at 1 or 500 mg kg^−1^ reduced nitrification activity by approximately 40% after 90 days. Ammonia-oxidising archaeal abundance decreased by 53% and 60%, respectively, demonstrating that prolonged exposure may cause substantial effects even at environmentally relevant concentrations [85]. Similarly, ZnO nanoparticles applied at 0.5–2.0 mg g^−1^ reduced bacterial populations by 36–48% in saline–alkaline soil, whereas black soil was less affected, demonstrating the importance of soil type [86].

Plant responses are also concentration- and model-dependent. In wheat, ZnO toxicity was greater in acidic soil, where soluble Zn and shoot Zn concentrations were approximately 200-fold and tenfold higher, respectively, than in alkaline soil [87]. In soybean seedlings, 8 nm ZnO nanoparticles increased root growth by 30% at 500 mg L^−1^ but reduced it by 40% at 4000 mg L^−1^. Zinc accumulation reached 229 mg kg^−1^ dry weight at 500 mg L^−1^, while ZnO was transformed into Zn(II) species within root tissues [88]. Conversely, TiO_2_ nanoparticles at 2–10 mg L^−1^ promoted wheat seedling growth, whereas higher concentrations produced inhibitory or negligible effects [89]. These findings demonstrate that nanoparticle effects may be beneficial at low concentrations but toxic at higher concentrations.

Nanoparticle biodistribution also depends strongly on the exposure route. In minipigs treated with 5% TiO_2_ sunscreen for four weeks, particles were predominantly retained within the stratum corneum and hair follicles, with no increased titanium detected in the liver or lymph nodes [90]. In a human study, five-day application of sunscreen containing 19 nm ZnO produced only a small increase in zinc tracer in blood, approximately 0.1% of total blood zinc, although whether this represented intact nanoparticles or dissolved zinc was unclear [91]. Collectively, these findings indicate limited systemic distribution through intact skin but highlight the importance of particle dissolution, skin condition, dose and exposure duration.

### 3.11. Translational and Regulatory Considerations

Beyond biological safety, successful implementation requires scalable manufacturing, reproducible synthesis, and regulatory compliance. One of the most significant challenges is poor reproducibility, driven by variability in plant extract composition, phytochemical content, extraction methods, precursor chemistry, and synthesis conditions. Small changes in these parameters can substantially alter nanoparticle morphology, surface chemistry, ROS behaviour, and biological interactions [10,33]. Regulatory approval is particularly challenging because hybrid bio-nano systems do not fit neatly within existing cosmetic, pharmaceutical, or medical-device classifications. In addition, standardised characterisation methods and benchmarking protocols remain insufficiently developed across the field. As a result, direct comparison among studies is often difficult [16].

For sunscreen and cosmetic applications, regulatory approval requires substantially more than demonstration of UV-blocking efficacy [1,13]. Candidate nanomaterials must undergo comprehensive physicochemical characterisation, including particle size distribution, morphology, surface chemistry, crystallinity, zeta potential, aggregation behaviour, and batch-to-batch reproducibility [6,13,15]. Functional performance should be evaluated using recognised SPF and UVA protection methods together with assessment of photostability following UV exposure [2,13]. In addition, safety evaluation should include dermal penetration studies, skin irritation and sensitisation testing, cytotoxicity, genotoxicity, and photo-induced toxicity [13,58]. Increasing regulatory attention is also being directed towards the environmental fate and ecotoxicological effects of nanoparticles released from cosmetic formulations. Collectively, these requirements highlight that successful clinical and commercial translation depends not only on photoprotective efficacy but also on robust manufacturing, reproducible product quality, comprehensive safety assessment, and regulatory compliance [1,13,16].

Another limitation is the heavy reliance on simplified in vitro biological models [13,16]. Many studies fail to adequately evaluate long-term exposure, chronic inflammation, immune responses, skin barrier interactions, environmental release, or realistic formulation conditions [1,16]. More physiologically relevant models, including reconstructed human skin systems and long-term in vivo studies, will therefore be necessary [1,16]. Scalable manufacturing also remains challenging because biological raw materials exhibit inherent compositional variability. Bridging the gap between laboratory synthesis and industrial production will require improved process standardisation, quality-control frameworks, and interdisciplinary collaboration among nanotechnologists, dermatologists, toxicologists, materials scientists, and regulatory agencies [1,15].

## 4. Future Perspectives

Despite significant progress, several challenges remain before plant-derived nanomaterials can be fully translated into practical and commercial photoprotective technologies. One of the major priorities for future research is the establishment of standardised, scalable, and reproducible green synthesis protocols. Variability in plant extract composition, synthesis conditions, and nanoparticle characteristics can influence the physicochemical properties and biological performance of the resulting nanomaterials. Integrating metabolomic characterisation of plant extracts with machine learning-guided optimisation may help identify the phytochemicals responsible for nanoparticle formation and improve manufacturing consistency. Machine learning offers the opportunity to correlate synthesis parameters (plant species, extract composition, pH, precursor concentration, and reaction temperature) with nanoparticle size, crystallinity, zeta potential, bandgap, and photoprotective performance, enabling predictive optimisation rather than empirical trial-and-error synthesis [92,93]. Similar predictive models could be applied to plant-mediated photoprotective nanomaterials to optimise UV absorption, minimise photocatalytic ROS generation, and improve batch-to-batch reproducibility.

Another critical area of investigation involves the comprehensive evaluation of safety and long-term biological effects. Although plant-mediated nanoparticles are generally considered biocompatible, detailed studies addressing skin penetration, cytotoxicity, immune responses, and long-term exposure are required to validate their safety for widespread cosmetic and biomedical use. In parallel, regulatory frameworks and testing standards must evolve to address the growing use of bio-derived nanomaterials in consumer products. Future progress will likely depend on transitioning from empirical material discovery to predictive and design-driven engineering approaches that integrate computational modelling, high-throughput synthesis, and systems-level biological evaluation. Innovations such as hybrid bio-nanocomposites, surface-engineered nanoparticles, stimuli-responsive materials, and controlled-release antioxidant platforms could significantly enhance UV shielding efficiency while improving stability and durability in practical applications. Interdisciplinary collaboration among materials scientists, chemists, dermatologists, and biomedical researchers will be crucial to accelerating progress in this field. Ultimately, continued research and technological innovation may enable plant-derived nanomaterials to serve as the basis for next-generation sustainable photoprotective solutions, contributing not only to improved skin health but also to the development of environmentally responsible materials.

## 5. Conclusions

Plant-mediated photoprotective nanomaterials combine UV attenuation with antioxidant and anti-inflammatory functions. Their performance depends on the interaction of optical properties, ROS regulation, surface chemistry and biological interfaces. However, comparison and translation remain constrained by variability in material composition, synthesis conditions and experimental models.

Future progress requires standardised manufacturing and evaluation, predictive structure–function engineering, rigorous long-term safety assessment and multifunctional hybrid designs. Addressing these priorities may enable safer and more sustainable photoprotective technologies.

## Figures and Tables

**Table 1:** Comparison of conventional UV filters and plant-derived nanomaterials. Plant-mediated nanoparticles are synthesised using plant extracts, whereas herbal nanocomposites are composite materials that incorporate plant-derived bioactive compounds with nanomaterials to enhance photoprotective performance.

Category	Representative Materials	Size (nm)	Mechanism	UV Coverage	ROS Behaviour	Advantages	Limitations	Translation
Organic UV filters	Oxybenzone, avobenzone	≤1 nm	UV absorption via molecular chromophores	UVA and/or UVB (compound-specific)	Minimal intrinsic ROS generation	Transparent, easy formulation, widely used	Photodegradation, potential toxicity, and environmental concerns	High (commercially established) [23,67].
Inorganic nanoparticles	ZnO, TiO_2_	~10–100	UV absorption and scattering (bandgap-dependent)	Broad-spectrum (ZnO: UVA + UVB; TiO_2_: mainly UVB)	Can generate ROS under UV irradiation	High stability, strong UV-blocking efficiency	Potential oxidative stress, aggregation, and safety concerns at the nanoscale	High (widely used in sunscreens) [14,23].
Plant-mediated nanoparticles	Plant-extract synthesised ZnO, Ag and Au	10–150	Combined UV attenuation + phytochemical-mediated antioxidant activity	Broad-spectrum (material-dependent)	Reduced ROS due to phytochemical capping; antioxidant activity	Eco-friendly synthesis, improved biocompatibility, and multifunctionality	Batch variability, limited standardisation, and scalability challenges	Moderate (emerging) [12].
Plant-derived organic nanoparticles	Lignin, cellulose, phytopolymer nanoparticles	50–300	UV absorption + intrinsic antioxidant activity	Primarily UVB, partial UVA	ROS scavenging dominant	Biocompatible, biodegradable, low toxicity	Lower UV-blocking efficiency	Low–moderate [24,28,37].
Herbal nanocomposites	Lignin–ZnO, chitosan-based composites	50–200	Synergistic UV absorption, scattering, and antioxidant activity	Broad-spectrum	Balanced ROS generation and scavenging	Enhanced stability	Limited regulatory approval, complex synthesis	Moderate [29,65].
Phytochemical-loaded nanocarriers	Curcumin, resveratrol, catechin-loaded systems	50–200	Indirect photoprotection via ROS scavenging and signalling modulation	Indirect (not primary UV blockers)	Strong ROS scavenging	Improved bioavailability, targeted delivery	Limited direct UV-blocking capability	Low–moderate [30,31,32].

## Data Availability

No new data were created or analyzed in this study. Data sharing is not applicable to this article.
